# The case for targeting latent and lytic Epstein-Barr virus infection in multiple sclerosis

**DOI:** 10.1093/brain/awaf170

**Published:** 2025-05-06

**Authors:** Gavin Giovannoni, Louisa James, Adekunle A Adeniran, Julian Gold, Lawrence S Young, David L Selwood, David Baker, Ruth Dobson

**Affiliations:** Faculty of Medicine and Dentistry, Blizard Institute, Queen Mary Univesity of London, London E1 2AN, UK; Faculty of Medicine and Dentistry, Blizard Institute, Queen Mary Univesity of London, London E1 2AN, UK; Faculty of Medicine and Dentistry, Blizard Institute, Queen Mary Univesity of London, London E1 2AN, UK; Faculty of Medicine and Dentistry, Blizard Institute, Queen Mary Univesity of London, London E1 2AN, UK; The Albion Centre, University of Sydney, Sydney, NSW 2010, Australia; Warwick Cancer Research Centre, Warwick Medical School, University of Warwick, Coventry CV2 2DX, UK; Wolfson Institute for Biomedical Research, University College London, London WC1E 6JL, UK; Faculty of Medicine and Dentistry, Blizard Institute, Queen Mary Univesity of London, London E1 2AN, UK; Faculty of Medicine and Dentistry, Wolfson Institute of Population Health, Queen Mary University of London, London EC1M 6BQ, UK

**Keywords:** multiple sclerosis, Epstein-Barr virus, antiviral therapies

## Abstract

Epstein-Barr virus (EBV) is strongly associated with multiple sclerosis (MS). It is likely to play a causal role in the pathogenesis of MS, possibly via triggering autoimmunity through molecular mimicry, autoantigenic presentation or immune dysregulation. Alternatively, evidence supports a direct role for EBV in driving MS disease activity via latent-lytic infection cycling either within the CNS or the periphery. We highlight the recent immunological and virological findings supporting the role of active EBV infection in MS, supporting an evaluation of anti-EBV strategies as potential treatments for MS.

Anti-EBV strategies include CNS penetrant small molecule anti-viral agents targeting latent and lytic infection, and immunotherapies. Immunotherapies include EBV-specific autologous or allogeneic cytotoxic T cells (CTLs) and therapeutic EBV vaccines and/or immune checkpoint inhibitors to rejuvenate and boost endogenous EBV-targeted CTL responses. In parallel, several licensed MS disease-modifying therapies may work via mechanisms targeting EBV directly or indirectly. B-cell depleting therapies have been shown to have anti-EBV activity; additionally, new strategies to target intrathecal B cells, plasmablasts and plasma cells are being explored, including high-dose anti-CD20 therapy, cladribine, proteasome inhibitors, BTK inhibitors, CNS-penetrant anti-CD20/CD19 monoclonal antibodies and CD19-targeted CAR T cells. Innovative trial designs for proof-of-concept studies to test EBV antivirals and immunotherapies in MS are needed to catalyse a wave of drug development targeting EBV as a therapeutic strategy to prevent or treat MS.

## Teaser

Epstein-Barr virus (EBV), which causes infectious mononucleosis, may play a causal role in multiple sclerosis by altering immune system function. Giovannoni *et al.* highlight recent findings that support a role for active EBV infection in multiple sclerosis and emphasize the need to explore treatments targeting EBV.

## Introduction

**Figure 1:**
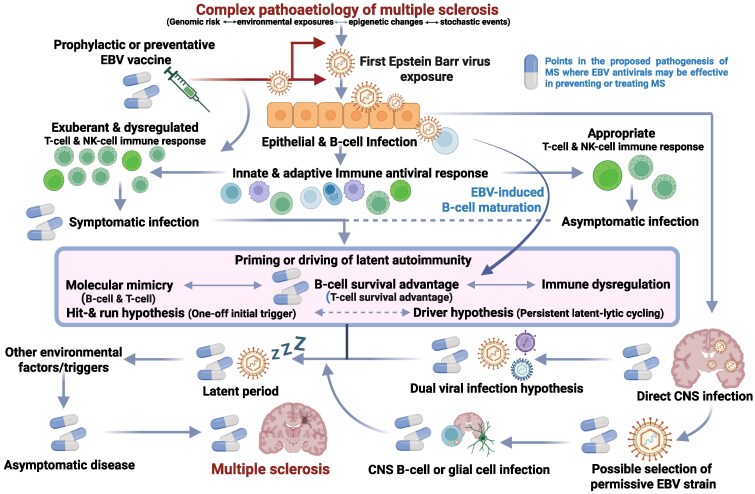
**Role of Epstein-Barr virus in the pathogenesis of multiple sclerosis.** A simple schematic of the potential role Epstein-Barr virus (EBV) plays in the pathogenesis of multiple sclerosis (MS) and at which points EBV latent-lytic cycling may be targeted with EBV antivirals to prevent or treat MS. Please note that if EBV is triggering MS disease activity in the CNS, then CNS-penetrant antivirals or EBV immunotherapies will be required. This figure has evolved from a previously published editorial commentary.^5^

Epstein-Barr virus (EBV) almost certainly plays a causal role in the aetiology of multiple sclerosis (MS), being necessary but insufficient for someone to develop MS.^1,2^ EBV-negative people are protected from getting MS; their risk is close to zero.^3^ Studies of EBV-negative people who develop MS in the future demonstrate that they seroconvert to EBV-positive prior to the clinical onset of MS, which is not seen with other viruses.^4^ However, despite strong epidemiological evidence linking EBV to MS development, a mechanistic understanding of how EBV might cause MS is lacking. Current theories include molecular mimicry, via virally expressed antigens, autoreactive B-cell immortalization, immune dysregulation, active EBV viral infection or activation of a downstream causative virus (or viruses), such as human endogenous retroviruses (dual-virus hypothesis). Whether EBV simply triggers MS and then does not play a role in disease pathogenesis (hit-and-run hypothesis) or causes and continues to drive the disease process via active infection (the driver hypothesis) is unknown^5^ (Fig. 1). However, as MS is typically relapsing, this suggests the presence of a cycling risk factor.

Despite the uncertainty about how EBV causes MS, epidemiological and other observations support the case for testing anti-EBV strategies both as a potential preventive intervention and as potential disease modifying agents for established MS. There is a need to consider how a population-based EBV vaccine programme aimed at preventing primary EBV infection and EBV-related infectious mononucleosis might be harnessed to understand the impact on development of MS and other EBV-related diseases such as Hodgkin's lymphoma and other EBV-related autoimmunities.^1,2^ Studying this relationship is challenging for practical reasons. There is a long lag between primary EBV infection and MS diagnosis; the relative rarity of MS combined with the near ubiquity of EBV infection and asymptomatic seroconversion in many are a few of the barriers to high-quality prospective studies. In this review, we discuss potential mechanisms via which EBV might cause and drive ongoing disease activity in MS and the antiviral strategies that could be studied as part of MS prevention and treatment.

## Molecular mimicry

Molecular mimicry is currently the favoured hypothesis amongst immunologists to explain how EBV might cause MS.^6-11^ Molecular mimicry implies that EBV antigens generate cross-reactive immune responses to self-proteins/antigens that trigger an immune attack on self, which in the case of MS is the myelin-axon unit (Table 1). Several autoantigens have been shown to have cross-reactive immune responses between self-epitopes (autoantigens) and EBV proteins at the T- and B-cell levels.^6-9,11^ However, many of these antigens are not only expressed in the CNS but elsewhere challenging the molecular mimicry hypothesis. For example, glialCAM, a recent putative autoantigen, is expressed in the liver and present in hepatocytes, where it is referred to as HepaCAM.^27^ Further, in addition to being present in myelin, it is predominantly present on astrocytes within the CNS, with functional deficiency causing megalencephalic leukoencephalopathy with subcortical cysts. If glialCAM/HepaCAM were driving MS as an inflammatory autoantigen, one would expect comorbid autoimmune hepatitis and astrocytic pathology analogous to anti-aquaporin-4 associated neuromyelitis optica.^28^

**Table 1:** Putative autoantigens hypothesized to drive Epstein-Barr virus-associated molecular mimicry in multiple sclerosis

Putative autoantigen	EBV-protein
Myelin basic protein (MBP)	EBNA-1^12,13^
	BALF5^14-16^
	LMP1^17,18^
Anoctamin 2 (ANO2)	EBNA1^10^
Anoctamin 2 (ANO2)	BPLF1 and BHRF1^19,20^
Glial cell adhesion molecule (GlialCAM)	EBNA-1^11,21,22^
HLA-DR-derived self-peptides	BPLF1 and BHRF1^19,20^
αB-crystallin (CRYAB)	EBNA-1^23-26^

EBV = Epstein-Barr virus.

Although mimicry or shared epitopes can be found between infectious agents and autoantigens, this is not entirely unexpected, as T- and B-cell antigen receptors are highly promiscuous in their binding potential. However, despite the observation that every individual seems to harbour autoreactive cells, these do not necessarily drive MS; immune checkpoints and T regulatory cell networks exist to limit this activity to prevent memory cell activation. Importantly, immune tolerance to presumed mimicked epitopes has failed to alter MS significantly,^29^ implying that molecular mimicry alone is insufficient. A combination with acquired impairment of immune tolerance is also a necessary step. Many of the proposed autoantigens are intracellular, and many have sequences homologous with other viruses not associated with MS. These are typically identified in people with pre-existing damage, suggesting that any detected autoreactive may be secondary to the primary events.

One potential mechanism not requiring the complexity of cross-reactive immunity is following antigen-driven immune cell priming. Here, autoantigens, such as alpha B crystallin or CRYAB, arguably the immunodominant autoantigen in inflamed MS brain, which is absent in the thymus,^30^ escape thymic negative selection, allowing autoreactive naive cells to bypass immune tolerance and circulate into lymphoid tissues. Following subsequent EBV and other viral infections, CRYAB, a heat shock protein (HSPB5), is expressed and presented to generate memory CRYAB-specific cells. This biological process generates immune cells with lifelong (auto)immunity capable of migrating into peripheral tissues and the CNS. This can occur following endogenous and exogenous peripheral stimuli, such as infection, that may drive memory cells to enter the CNS and cause autoimmunity. Other events, such as infection and tissue damage, may also lead to oligodendrocyte stress and CRYAB expression within the CNS, resulting in amplification of inflammation with subsequent T- and B-cell autoimmunity. Inflammation as a means to allow protein entry is critical to the pathogenicity of peripheral autoreactive antibody responses.^23^ However, attempts to control disease by controlling this autoreactivity have so far failed.^31^

These inconsistencies and the observation that not one autoantigen is specific to MS, or a significant proportion of MS, cast some doubt over molecular mimicry being the primary mechanism of how EBV triggers and causes MS.

## Immune dysregulation

An alternative theory is that EBV infection causes dysregulation of the immune system, which is then primed to develop autoimmunity in the future; hence, EBV is necessary but insufficient to cause MS.

The second potential mechanism is viral-driven immune cell priming. EBV infection of naive B cells generates memory B cells. It has been proposed that latent EBV infection does this by providing infected B cells with a survival advantage, which allows putative autoreactive B cells to bypass endogenous tolerance mechanisms and thus have similar disease-induction potential as seen with antigen-driven immune cell priming^32^; however, evidence for this is lacking.^33^

The B-cell pro-survival signals come from EBV latent membrane proteins 1 and 2 (LMP1 and LMP2). LMP1 mimics CD40 signalling to activate multiple B-cell growth and survival pathways, particularly NF-κB.^34^ In parallel, LMP2A mimics the B-cell receptor (BCR) signalling cascade by bypassing the BCR and signalling directly through Syk/RAS/PI3K, Bruton’s tyrosine kinase (BTK) and STAT3, leading to increased IL10 production.^35^ IL10 then promotes B-cell proliferation and survival via autocrine mechanisms.^35,36^ In addition, EBV infection also induces CD40 ligand expression that can provide co-stimulatory activity.^37^

HLA-DRB1*15-01, a major MS-related genetic risk factor, promotes EBV viral load and infection in contrast to the HLA-A*02-01 resistance loci.^38^ This process could facilitate the development of other EBV-associated autoimmunities related to different HLA restrictions.^38,39^ DRB1*15 heterozygosity is predominant in systemic lupus erythematosus (SLE) patients and is associated with higher IgG titres to EBV early antigen (EA-D) and p22 and higher EBV viral loads compared to healthy controls.^38,39^ This supports the observation that EBV is not only linked to MS but several other autoimmune diseases as well; in particular SLE and potentially rheumatoid arthritis, Sjogren's syndrome, primary biliary cirrhosis, inflammatory bowel disease and several other autoimmune diseases, which harbour their own different MHC restrictions.^40,41^ In addition, memory B cells appear to be targeted to a greater or lesser extent by all effective MS treatments.^42^ Therefore, eliminating or controlling late-onset EBV infection has implications far outside the field of MS.

### Latent-lytic EBV infection and dual viral hypotheses

The molecular mimicry and immune dysregulation theories imply that the role of EBV in causing MS is like a hit-and-run accident; once autoimmunity is primed, targeting EBV will have no further impact on the development or course of MS, which is then driven by other factors unrelated to EBV, for example, genetic and other environmental factors.^43^ This is not necessarily correct, as intermittent EBV replication or latent-lytic infection cycling intermittently but continuously exposes the immune system to EBV antigens, which may be necessary to drive ongoing inflammatory disease activity via antigenic spread and immune dysregulation. As shown in mice, once sufficient priming has occurred to generate sufficient immune memory, environmental stimuli such as infection or infection-related superantigen may be sufficient to trigger memory cell circulation and initiate disease.^44,45^

### Driver hypothesis

EBV has been proposed as the ‘driver of MS’ by continually cycling through its latent and lytic infection phases.^46^ This could either be by (i) direct CNS infection; (ii) continuously stimulating autoreactive T and B cells; or (iii) upregulating a second virus such as MS-associated HERVs (human endogenous retroviruses or human herpes virus 6), which in turn cause tissue damage, including CRYAB production.^47^ The latter is the ‘dual-viral hypothesis’ of MS, whereby EBV acts upstream of a second virus^48^ (Fig. 1).

#### Dual viral hypothesis

The case for a second virus is probably most robust for two HERVs of the HERV-W family associated with MS. MS-associated retrovirus (MSRV) and ERVWE1, whose Env proteins are pathogenic *in vitro* and in animal models.^49^ Several studies have demonstrated weak associations between MSRV-Env and MS. MSRV expression in the CSF correlates with clinical worsening and the prognosis of MS.^50^ In post-mortem MS brain tissues, MSRV-Env protein localizes to MS plaques with a higher protein expression in active plaques compared to inactive plaques.^51,52^ MSRV-Env activates toll-like receptor 4 (TLR4), with pro-inflammatory effects.^53^ MSRV-Env can potentially disrupt oligodendrocyte differentiation during remyelination, which is also thought to be mediated via TLR4, expressed on oligodendrocyte precursor cells (OPCs).^54^ These observations prompted a phase 2 clinical trial of GNbAC1 or Temelimab, an IgG4 humanized monoclonal antibody against HERV-W-Env.^55^ Temelimab failed to show an effect on markers of acute MS inflammation, but study subjects treated with high-dose temelimab had fewer new T1-hypointense lesions (black holes) and a trend towards a reduction in brain atrophy and a decrease in magnetization transfer ratio (MTR) compared with the placebo group.^56^ The latter suggests a possible neuroprotective effect of temelimab. The better prognosis of people with MS with a genetic variant that can suppress HERVs more efficiently^57^ supports the HERV and dual viral hypotheses of MS.^47^ As EBV is hypothesized to work upstream of HERVs, suppressing EBV will prevent the downstream events due to HERVs.

Consistent epidemiological findings from England,^58^ Denmark,^59^ Sweden and Canada^60^ have shown that people who are HIV positive are at lower risk of developing MS. These observations have been made in the era of highly active antiretroviral therapy (HAART), suggesting that HAART and not HIV is responsible for this protection.^47^ In parallel, there are numerous case studies of people with comorbid HIV infection and MS in which starting HAART has coincided with remission of MS disease activity.^61-68^ As some antivirals in HAART are active against EBV^69^ and HERVs,^70^ the apparent therapeutic effect of HAART could be due to its suppression of EBV, HERVs or both. These observations underpin the rationale for testing antiretrovirals in MS (Supplementary Table 1).^71^

Human herpesvirus 6A (HHV-6A) is associated with an increased risk of developing MS.^72^ A recent Swedish nested case-control study linking the Swedish MS with Swedish biobanks showed that cases seropositive for HHV-6A had significantly higher levels of serum neurofilament light chain (sNFL) levels than controls prior to MS diagnosis in the asymptomatic or prodromal phase of MS.^73^ As seroreactivity against HHV-6A was detectable several years before the rise of sNfL the investigators suggest that HHV-6A infection may contribute to MS development in a proportion of cases as on only 40% of MS cases were HHV-6A seropositive compared to 25% of matched controls subjects.^73^ In this study, sNfL levels were significantly higher in both HHV-6A seropositive and seronegative cases compared to controls, indicating tissue injury was also occurring in the individuals who were HHV-6A negative.^73^ Serum NfL levels were even higher in subjects seropositive for both EBV and HHV-6A, indicating the two viruses are likely to act synergistically.^73^ Both EBV and HHV-6 and herpes viruses, in general, are known to transactivate HERVs and increase their expression, which could drive inflammation.^74^ Whether this is how EBV and HHV-6 interact requires further study. The association between HHV-6 and MS is not as consistent as EBV. It would not fulfil the Bradford-Hill criteria for causation, but HHV-6 could be a cofactor augmenting EBV as a driver of MS disease activity.^73^

#### Lymphoblastoid cell lines and salivary shedding

Another indicator that people with MS control EBV poorly is the observation that they are more likely to produce spontaneous EBV-associated lymphoblastoid cell lines (sLCLs) when their peripheral blood cells are cultured *ex vivo*,^75-77^ and people with MS, including children, appear to shed EBV in the saliva more frequently.^78-80^

It has also been shown that sLCLs derived from MS patient B cells during active disease have higher EBV lytic gene expression than sLCLs from MS patients with stable disease or healthy controls.^81^ People with MS have also been shown to have expanded T lymphocytes in the CSF that are specific for LCLs.^82^ These observations support the MS latent-lytic EBV infection hypothesis, albeit indirectly, and may indicate that the CNS is where this may be occurring. The observation that people with MS shed EBV in their saliva can potentially be used for proof-of-concept studies to assess whether EBV-target antivirals or immunotherapies can prevent latent-lytic cycling in people with MS in the systemic compartment. A simple cross-over design allows comparison of EBV shedding before, during and after antiviral therapy. Using this trial design, a recent study of famciclovir was shown not to suppress or reduce EBV shedding in the saliva of natalizumab-treated people with MS.^83^

#### Immunology

**Figure 2:**
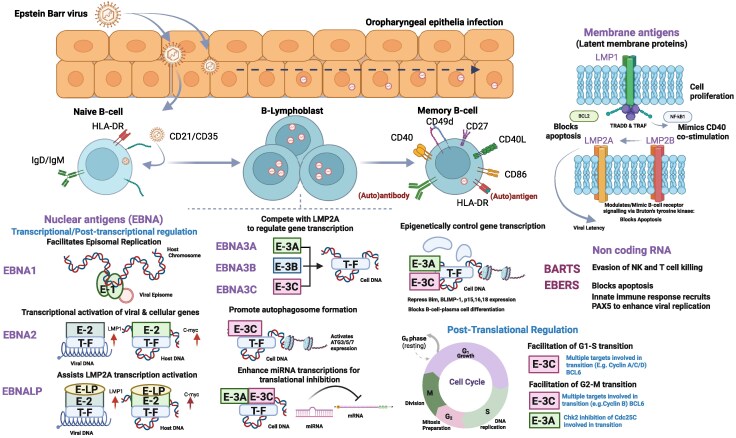
**Effects of latent Epstein-Barr virus proteins on B-cell function.** This figure is adapted from Saha and Robertson^87^ and summarizes some of the effects of latent Epstein-Barr virus (EBV) antigens on B-cell function. After initial infection of pharyngeal epithelial cells EBV primarily infects naïve B cells. Subsequently, the infected B cells transformed and express the full repertoire of latent EBV antigens. EBNA-1 (Epstein-Barr virus nuclear antigen-1) is responsible for attaching the EBV episome to the B-cell chromosome. EBNA-1 is important for both viral replication and segregation. EBNA-2 regulates viral and cellular gene transcription via RBP-Jκ (recombination signal binding protein for immunoglobulin-kappa J region). EBNA-LP (EBNA leader protein) promotes EBNA-2-mediated gene transcription. EBNA-3 proteins (-3A, -3B, -3C) block EBNA-2 association with RBP-Jκ, modulate viral gene transcription as well as the Notch signalling pathways. EBNA-3C forms a ternary complex with p53 and Mdm2 (mouse double minute 2 homologue) and recruits the Mdm2 E3 ligase for its degradation. EBNA-3C inhibits p53-dependent apoptosis and binds to and enhances the kinase activity of both cyclin D1/CDK6 (cyclin-dependent kinase-6) and cyclin A/CDK2 complexes to phosphorylate pRb (retinoblastoma protein). EBNA-3C also recruits SCFSkp2 E3 ligase activity on hyperphosphorylated pRb and p27KIP1 for degradation, thereby facilitating the transition of G1 to S phase. LMP-1 (latent membrane protein-1) inhibits p53-mediated apoptosis by transactivating Bcl-2 and A20 genes and augments NF-κB signalling to transactivate numerous cellular genes via interaction with TNF receptor associated factors (TRAFs) and TRADD. LMP-1 also modulates JAK/STAT, ERK/MAPK, IRF and Wnt signalling pathways and mimics CD40 signalling. LMP-2A blocks B-cell receptor signalling through interaction with cellular tyrosine kinases Lyn and Syk and signals via BTK (Bruton’s tyrosine kinase). LMP-2B regulates LMP-2A function. EBV-encoded small RNAs (EBERs) block apoptosis, bind to La and L22 cellular proteins and induce IL-10 cytokine and type-I IFN. This figure was created in BioRender. Baker, D. (2024) https://BioRender.com/e81t232.

The memory B cell is where EBV resides in its latent phase and is the seat of virus persistence.^84^ From a molecular perspective, EBV uses complex transcriptional programs to hibernate in B cells and potentially other cells, thereby evading the immune system^84-86^ (Fig. 2). Intermittent triggering of the replicative infection cycle either in the B-cell compartment or in epithelial cells lining the mouth and nasopharynx (latent-lytic cycling) allows the virus to spread from the host to new uninfected people via the transfer of infectious virus in saliva and genital fluids and rarely iatrogenically through blood and organs via transfusion and transplantation,^88^ thereby ensuring the virus survives from an evolutionary perspective.

While both latent and lytic EBV infection stimulates a humoral and cellular immune response to the virus, the antibody and T-cell response is dominated by recognition of EBV replicative antigens. The immune response to EBV latent proteins is muted with the low-level expression of the EBNA1 protein in persistently infected memory B cells able to evade cytotoxic T-cell recognition.^85^ The immune response to EBV infections involves innate^89-91^ and adaptive responses.^91-93^ Stimulating old memory or recall responses and developing new memory responses to EBV antigens and epitopes is analogous to the booster response to a vaccine.^94^

There is evidence that people with active MS have peripheral^95-97^ and CNS activation of the innate immune system.^98^ Innate immune activation can be measured using biomarkers, i.e. gene signatures in cells in the peripheral blood and/or brain or by measuring metabolites and cytokines of innate immune cells in the blood, urine and spinal fluid. These markers are raised in untreated people with MS who have active MS. One particular signature is the so-called type 1 interferon response in people with active MS and SLE.^99^ A proportion of patients with MS have a type 1 interferon response detectable in their peripheral blood transcriptome,^100^ which predicts poor treatment response to interferon-beta.^101-104^ As this response typically occurs in response to viral infections, these observations raise the question of whether or not the peripheral type 1 interferon response in untreated patients with active MS is a biomarker of EBV lytic infection.

Interestingly, fibroblast interferon or interferon-beta was initially administered intrathecally to patients with MS because of its antiviral activity and the hypothesis that MS was due to a viral infection.^105^ The baseline peripheral blood type 1 interferon gene signature predicts a poor therapeutic response to interferon-beta,^101-104^ implying that augmenting this type 1 interferon response therapeutically via the administration of exogenous interferon-beta is ineffective at controlling MS disease activity.

In parallel with innate immune changes, antibody (B cells) and T-cell responses to EBV can be measured in people with MS. People with MS have much higher levels and broader antibody responses to both latent^106^ and lytic^107^ EBV proteins. These immunological data suggest that people with MS have a problem suppressing EBV or maintaining it in its latent state. In addition to elevated antibody titres against the EBNA complex and EBNA1 antigen,^106^ people with MS have more EBNA1 reactive CD4+ T cells,^108^ which respond to a more extensive repertoire of epitopes distributed across the EBNA1 protein.^108^ In comparison, T cells from healthy controls only react to the immunodominant portion of the protein.^109^

A recent *in silico* analysis has shown that people with MS had more EBV-reactive T cells against EBNA1 and more clones of cells responding to a broader array of epitopes in the EBNA1 protein compared to control subjects and people with other diseases.^108,110^ The T-cell receptor repertoire analysis included identical twins. Twins concordant with having MS had a more expansive T-cell repertoire than those discordant with the disease.^110^ The latter monozygotic twin comparison avoids most of the confounding by genetic factors.^110^ It implies that these results are likely to be specific to having MS. Finding increased T cells reactive to both latent and lytic EBV antigens supports the hypothesis that latent-lytic EBV cycling is boosting T-cell memory and diversity in people with MS and strengthens the case for testing EBV antiviral agents in MS.^110^ In this study, people with MS treated with natalizumab had amplified T-cell responses to EBV; i.e. they had an even broader EBV reactive T-cell repertoire.^110^ Interferon-beta, a putative antiviral and anti-CD20 treatment targeting latent EBV in B cells, did not appear to modulate EBV-specific T cells.^110^

In control subjects without MS, EBV-specific T cells consist mainly of effector-memory cells in peripheral blood and CSF.^110^ In contrast, in people with MS, the CSF also contained EBV-specific central-memory T cells.^110^ This indicates recent priming of these cells, i.e. they had recently been exposed to EBV antigens, which in the case of CSF cells is likely to have taken place within the CNS of people with MS. These latter results are supported by another study showing expanded T lymphocytes in the CSF of people with MS specific for EBV-infected B cells.^82^

The observation that interferon-beta did not affect the anti-EBV T-cell responses in people with MS^110^ would argue against EBV latent-lytic cycling. However, it is important to note that interferon-beta is not a very effective disease-modifying therapy (DMT) and may not impact the CNS compartment where viral replication could occur.^111^ In comparison, ocrelizumab (anti-CD20) depletes memory B cells, including latent EBV infected B cells, and is a highly effective anti-inflammatory DMT, suppressing the majority of relapses and new MRI lesions.^112-115^ However, like interferon-beta, ocrelizumab does not affect EBV-targeted T-cell responses.^110^

However, in a follow-up longitudinal study, they demonstrate that ocrelizumab, teriflunomide or dimethyl fumarate, licensed MS DMTs, reduce EBV-specific, but not CMV-specific, MHC-I-restricted TCRβ sequences,^116^ implying that these agents may be working by suppressing EBV viral activity, i.e. latent-lytic cycling of the virus.

This evidence suggests that latent-lytic EBV cycling occurs in a compartment unaffected by anti-CD20 mediated peripheral B-cell depletion. Could this compartment be the CNS? Anti-CD20 monoclonal antibodies penetrate the CNS poorly and are unlikely to affect EBV-infected B cells within the CNS to a significant degree.^117,118^ In general, antibodies penetrate poorly into the CNS due to their size^119^ as exemplified by the anti-LINGO antibody Li81, which reaches CNS concentrations of only 0.1%–0.4% of blood levels.^120^

#### Anti-B cell activity in licensed MS disease-modifying therapies

Latent-lytic EBV infection and the dual viral hypotheses are why we should not assume that targeting EBV with either antivirals or immunotherapies after someone has developed MS will not work as a therapeutic strategy. The main reservoir for latent EBV virus is the memory B cell. Therefore, therapeutically targeting memory B cells will affect B-cell function and possibly EBV biology. If EBV is driving MS disease activity, then one could ask how licensed MS DMTs work in terms of EBV biology. The observation that all licensed DMTs in MS either reduce memory B-cell numbers or stop memory B cells from trafficking into the CNS^42,121-123^ supports this hypothesis. A possible exception to the memory B-cell hypothesis is teriflunomide. However, leflunomide, a prodrug that is rapidly converted into teriflunomide *in vivo*, inhibits B-cell function by decreasing B-cell proliferation and progression through the S phase in a non-selective manner.^124-126^

In patients with rheumatoid arthritis treated with leflunomide, the percentage of circulating memory B cells within the CD19+ pool does not necessarily change; however, the number of circulating CD19+ B cells is reduced due to drug treatment. Therefore, leflunomide and, by inference, teriflunomide decrease the absolute number of memory B cells.^126^ However, the decrease in memory B cells differs quantitatively from high-efficacy B-cell depletion agents such as anti-CD20 therapies. Plasmablasts are particularly affected by leflunomide and teriflunomide.^126,127^ Teriflunomide exhibits a similar effect in patients with MS compared to patients with rheumatoid arthritis on leflunomide, whereby the percentage of circulating memory B cells does not change.^127,128^ However, in some instances, the absolute number of CD19+ B cells decreases, significantly reducing circulating memory B-cell numbers.^128,129^ In addition, leflunomide and teriflunomide have pan-antiviral activity and may work in MS by inhibiting lytic EBV infection.^130-133^

As a corollary, atacicept,^134^ which neutralizes BAFF (B-cell activating factor) and APRIL (a proliferation-inducing ligand), and anti-TNF-alpha (infliximab and lenercept),^135^ which have both been shown to exacerbate MS disease activity, are known to expand peripheral memory B-cell numbers.^42^ Memory B cells as a therapeutic target may be critical in explaining the effects of therapeutic agents in MS.

#### Lessons from natalizumab

Natalizumab does not deplete peripheral immune cells as it is a selective adhesion molecule inhibitor, which targets VLA4 (very late antigen-4) or α4β1 integrin, the ligand for VCAM-1 (vascular cell adhesion molecule-1). By blocking the VLA4–VCAM-1 interaction, natalizumab prevents the trafficking of lymphocytes and monocytes into the CNS.^136^ As natalizumab does not affect the observed T-cell responses to EBV in people with MS, it suggests this latent-lytic cycling continues unabated, and natalizumab is blocking the migration of these T cells into the relevant tissue or end-organ to mount an immune response to EBV. This data^110^ does not tell us whether the EBV latent-lytic cycling occurs within the CNS or peripheral compartment. However, EBV antigens from CNS lytic infection will likely reach the periphery via interstitial fluid and CSF outflow pathways and stimulate peripheral T- and B-cell responses.

#### JC virus and progressive multifocal leukoencephalopathy

JC virus (JCV) lytic infection in progressive multifocal leukoencephalopathy (PML) in natalizumab-treated patients allows JCV-associated antigens to reach the periphery via interstitial fluid and CSF outflow pathways and stimulate peripheral T- and B-cell responses. A rising JCV antibody index, or titre, presages the overt development of PML in natalizumab-treated people with MS.^137^ The immunological data tell us that JCV is active and replicating, and by doing this, it is boosting the immune response to its viral proteins. The compartment the JCV replicates in is likely the CNS. The strain of JCV that causes PML is a mutant strain that acquires the ability to infect glial cells.^138^ An evolutionary process selects for mutations that improve the JCV's fitness while allowing it to escape detection by the immune system.^139^ Mutant strains out-compete non-mutant strains because of their ability to infect glial cells and survive. Mutant PML-causing JCV strains almost certainly need to replicate in the presence of glial cells to drive their evolution. Could the same process be happening with EBV within the CNS? This is possible, but the evidence that EBV infects neurons^140^ or glial cells^141^ is rudimentary and needs confirmation. However, the latent-lytic cycling hypothesis for EBV may occur in the intrathecal B-cell compartment and does not necessarily require additional cell types. In all likelihood, we will require CNS penetrant anti-EBV strategies to target these processes.

##### Natalizumab rebound

Two patients who died of rebound MS disease activity after natalizumab therapy was stopped were subsequently shown at post-mortem to have lytic EBV infection within their brains associated with a massive immune response against the virus and evidence of bystander damage (demyelination and axonal transection).^142,143^ Lytic infection in these two cases was detected using immunohistochemistry with antibodies against EBV late lytic protein p160 (OT10) and gp350/220 (OT 6.2).^142,143^ Therefore, these findings are unlikely false positives^142,143^ and raise the critical question of whether or not all natalizumab rebound is in response to CNS EBV lytic infection. If this proves true, then natalizumab would represent a forme fruste of the immune reconstitution inflammatory syndrome (IRIS), which was initially described in AIDS patients who developed brisk immune responses to opportunistic infections in the CNS with the initiation of antiretroviral therapy.^144^

The obvious question in relation to these two cases of EBV-associated natalizumab-rebound is how EBV got into their brains. Was EBV already present in the CNS, or was the virus carried into the CNS with the recommencement of memory B-cell trafficking after natalizumab was stopped and allowed to wash out? In support of the former is the increase in T-cell responses to both latent and lytic EBV antigens described in people with MS on natalizumab.^110^ This would indicate that people with MS on natalizumab are boosting their immune responses to EBV despite the cells being confined to the periphery, as they are prevented from trafficking into the target organ, i.e. the brain and spinal cord. In contrast to the T-cell responses, anti-EBNA-1 and anti-VCA (viral capsid protein) antibody titres do not increase in people with MS on natalizumab^145-147^ and argue against the CNS lytic infection hypothesis; one would expect anti-EBV antibody titres to rise analogous to what happens with anti-JCV antibody titres in people with MS who develop natalizumab-associated PML.^137^

##### Rituximab prevents natalizumab-rebound

It is worth noting that rituximab, an anti-CD20 therapy, is very effective in preventing natalizumab-associated rebound from occurring.^148^ As anti-CD20 targets mainly B cells, it is reasonable to assume that EBV-infected memory B cells carry the virus into the CNS. This is the so-called ‘Trojan horse hypothesis’. Why latently EBV-infected B cells would suddenly reactivate to cause massive lytic infection is unknown. It is clear that natalizumab rebound, as identified using Gd-enhanced MRI, is a multifocal phenomenon and occurs across the CNS. This would indicate that EBV viral reactivation must be coordinated across the CNS and occur in a relatively tight time window.^149^

The two patients with natalizumab rebound and lytic EBV infection had CD4+ and CD8+ T-cell infiltrates, with CD8+ T cells predominating.^142,143^ A small population of T cells express CD20, which they are thought to acquire from B cells through trogocytosis.^150^ Trogocytosis is when one cell physically extracts, ingests or bites off cellular material from another cell.^151^ In people with MS, it is thought that T cells acquire CD20 on their surface after interacting with B cells during the process of B-cell antigen presentation. This would imply that these CD20+ T cells are activated effector T cells and likely pathogenic (anti-EBV) and susceptible to depletion by anti-CD20 therapies.^150^ As anti-CD20 therapies deplete this population of CD20+ T cells, it could be argued that this population of T cells, and not necessarily B cells, are responsible for natalizumab rebound.

Natalizumab rebound is a potential model for testing EBV antiviral strategies in people with MS. If a CNS penetrant EBV-specific antiviral targeting lytic infection started before natalizumab wash-out prevents rebound disease activity, this would go a long way to supporting that EBV lytic infection is the cause of natalizumab rebound disease activity.

## Targeting EBV

**Figure 3:**
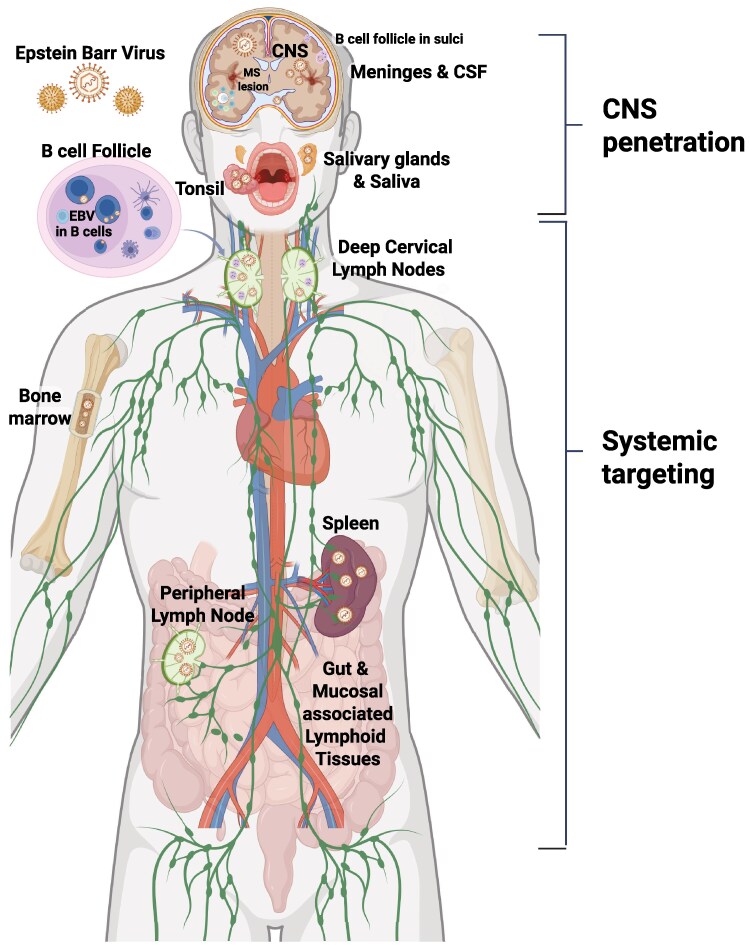
**Anatomical sites to target Epstein-Barr virus.** The potential sites where Epstein-Barr virus (EBV) resides that need to be targeted with EBV antivirals to treat multiple sclerosis. This figure was created in BioRender. Baker, D. (2024) https://BioRender.com/j57v337.

These analyses support the EBV driver hypothesis and argue for testing peripheral and CNS-penetrant anti-EBV strategies. EBV has several niches that need targeting (Fig. 3). These include EBV-specific antiviral agents and treatments targeting CNS resident B cells, such as cladribine, proteasome inhibitors, Bruton’s tyrosine kinase inhibitors (BTKi), CNS-penetrant anti-CD20 and anti-CD19 monoclonal antibodies and EBV-targeted and CD19-targeted CAR T cells^152^ (Supplementary Table 2). Cladribine is a CNS penetrant nucleoside analogue that is licensed to treat patients with relapsing MS. Cladribine reduces peripheral blood B cells and suppresses memory B cells for prolonged periods of time.^123^ It has been shown to reduce the number CSF oligoclonal IgG bands,^153^ and in a long-term follow-up over 50% of cladribine treated subjects lost their oligoclonal IgG bands.^154^ Proteasome inhibitors are licensed as adjuvant therapy for patients with myeloma and are used off-label to treat resistant antibody-mediated autoimmune diseases.^155^ CNS penetrant proteasome inhibitors may therefore target antibody producing cells. including B cells, plasmablasts and plasma cells; for example, ixazomib, a second-generation proteosome inhibitor, is currently being tested in MS (ClinicalTrials.gov ID NCT03783416). Covalent and non-covalent BTK inhibitors seem as a class to be effective against EBV-associated CNS lymphomas and post-transplant lymphoproliferatives disorders and reduce EBV viral loads.^156,157^ BCR signalling, which activates EBV lytic induction in freshly isolated B cells from peripheral blood mononuclear cells (PBMCs) can be inhibited by BTKi's ibrutinib or idelalisib.^158^ Several second-generation BTK inhibitors, some of which have been shown to be CNS penetrant, are in late-stage development as treatments for MS.^159^ One of the modes of action of BTKs may therefore be against EBV.

### EBV antivirals

Targeting EBV with antiviral drugs to stop latent lytic-cycling or purge the body of latent virus are obvious strategies that need exploration. We do not know if we need to target lytic EBV infection, latent EBV infection or both and in which compartment: the CNS, peripheral compartment or both. Based on the observation that people with MS cannot control EBV infection we suspect that we will need to target both lytic and latent cycles of EBV infection, and we will also need to use antivirals that work in the periphery, deep tissue and CNS. The immunological and pathological evidence, reviewed earlier, implicates the CNS, deep tissue and peripheral compartments (Fig. 3). In healthy individuals, EBV-specific T cells consist mainly of effector-memory cells in the peripheral blood and CSF.^110^ In contrast, in subjects with MS, the CSF contains EBV-specific central-memory T cells,^110^ indicating these cells are likely to have recently been primed or recently exposed to EBV antigens, which in the case of CSF cells is likely to have taken place in the CNS. These immunological data suggest antiviral strategies should target the CNS.

#### Purging EBV

One therapeutic strategy is to try to purge the body of latently infected B cells to reduce the viral load. This would also potentially remove the autoreactive antigen-presenting B cells driving MS. Purging B cells can be done using cell-depleting strategies, including immune reconstitution therapies (AHSCT, alemtuzumab, cladribine, mitoxantrone) and targeted B-cell depleting agents (anti-CD20 and anti-CD19 monoclonal antibodies), or by targeting the latent virus in EBV infected cells with cellular therapies and antivirals such as EBNA1 inhibitors,^160^ DNA hypomethylating agents that inhibit DNA methyltransferases (such as 5-azacytidine and decitabine) or HDAC inhibitors in combination with an EBV antiviral (for example, nanatinostat in combination with a nucleoside antiviral such as valganciclovir) (ClinicalTrials.gov Identifier: NCT05011058, Viracta Therapeutics). DNA methyltransferases such as decitabine, a potent inducer of immunogenic EBV antigens including LMP1, EBNA2, and EBNA3C, theoretically sensitize cells to lysis by EBV-specific cytotoxic T lymphocytes^161^ and could be used in combination with cell-based immunotherapies. This strategy is akin to what has been proposed in HIV, i.e. using treatment strategies to flush out a latent or hidden pool of the virus to allow the immune system to eliminate the virus.^162^ It is important to note that this strategy has not been successful in HIV.

#### Anti-CD20 and anti-CD19 therapies

Rituximab, a first-generation depleting anti-CD20 monoclonal antibody, is used extensively for EBV-associated lymphoproliferative disease and causes peripheral EBV viral loads to fall rapidly.^163^ Although rituximab depletes B cells in the peripheral blood, spleen and lymph nodes, it does not stop the salivary shedding of EBV.^164^ Whether the latter observation is because anti-CD20 therapy does not penetrate the salivary glands to deplete tissue-resident B cells or EBV resides in salivary gland epithelial cells that do not express CD20 is unknown. This observation implies that in the case of anti-CD20 and likely anti-CD19 targeted therapies, an EBV viral pool will persist, leaving a need for EBV-specific antiviral therapies to prevent reinfection of the reconstituted B-cell population. This underpins the rationale for using an anti-CD20 or anti-CD19 monoclonal antibody as induction therapy, followed by a maintenance therapy that prevents EBV lytic infection and the reinfection of the reconstituted B-cell population. Potential agents could include DHODH inhibitors (teriflunomide, leflunomide, vidofludimus, ASLAN003),^165^ HAART (highly active antiretrovirals), for example tenofovir^69^ and famciclovir,^166,167^ or another anti-EBV viral agent.^167^ If effective, this strategy has the advantage of derisking the chronic immunosuppression associated with long-term B-cell depletion, particularly the development of hypogammaglobulinaemia. This strategy allows people with MS to reconstitute their B-cell compartments, enabling them to respond to vaccines and deal with community-acquired and opportunistic infections associated with chronic B-cell depletion. Many antiviral drugs belong to the nucleoside or nucleotide class (tenofovir) and do not readily penetrate the CNS, so further development of antivirals will be required if CNS anti-EBV activity is necessary for adequate treatment of MS.^168^

As discussed earlier, if EBV drives MS disease activity from within the CNS, sufficient doses of anti-CD20 therapies are unlikely to penetrate the CNS to purge this compartment of EBV. Notably, anti-CD20-treated people with MS do not deplete B cells in their CSF compartment^117,118^ or lose B-cell associated oligoclonal IgG bands.^169^ Whether or not higher doses of ocrelizumab, a second-generation anti-CD20 depleting monoclonal antibody, will achieve this depends on the outcome of the ongoing phase 3 trials exploring 1800 and 1200 mg doses of ocrelizumab compared to the standard 600 mg dose (NCT04544436, NCT04548999).

Another potential option is the development and testing of CNS penetrant anti-CD20 or anti-CD19 antibodies using the transferrin receptor 1 (TfR1 or CD71) as a shuttle to get higher doses of antibody across the blood–brain barrier. This could include third-generation anti-CD20 antibodies that kill via apoptosis without the need for secondary effector systems.^170,171^

#### EBNA1 inhibitors

The Epstein-Barr nuclear antigen 1 (EBNA1) is chief among the latency genes and is consistently expressed in all EBV-positive tumours. EBNA1 is required to maintain the EBV genome in latently infected cells and provides a survival function to host cells.^172,173^ EBNA1 localizes to the nucleus and binds to circularized EBV episomal DNA at a specific region known as the latent origin of replication (OriP). In addition to its role in replication, EBNA1 binds to a viral region called the Family of Repeats (FR), while simultaneously binding to cellular genomic DNA. Thus, EBNA1 acts as a tether to promote the distribution of viral DNA to each of the daughter cells during cell division. EBNA1 also acts as a transcription factor for other latent EBV genes, including EBNA2 and LMP, and influences the expression of various host genes. Thus, EBNA1 plays a critical role in several processes that promote and maintain persistence and is an ideal target for pharmacological inhibition of EBV.

Small interfering RNA (siRNA) knockdown of EBNA1 in EBV-driven models and a dominant-negative version of EBNA1 causes substantial cell death and inhibition of cellular growth.^174-178^

Small molecule inhibitors of the DNA binding activity of EBNA1 have been developed. The efficacy of VK2019, an EBNA1 inhibitor, has been well studied in several EBV-dependent xenograft models^160,179^ and is currently being evaluated as a treatment in nasopharyngeal carcinoma (ClinicalTrials.gov Identifier: NCT04925544). Data from this latter study, particularly the pharmacokinetic profile of VK2019, will allow some prediction of the potential dose range for patients with MS. The favourable adverse events profile from this study suggests VK2019 will be well tolerated.^180^ Another approach uses a peptide inhibitor to disrupt the EBNA1 homodimer with the additional benefit of providing a luminescent signal to allow *in vivo* imaging.^181^ Modified versions of this peptide inhibitor have been shown to induce the EBV lytic cycle, thereby rendering infected cells more susceptible to T-cell recognition.^182^

### Immunotherapies

It has been argued that exhausted EBV-targeted T-cell responses, particularly the CD8+ cytotoxic T-cell response, lead to poor virus control, which drives MS disease activity.^183,184^ It has been proposed to use EBV-targeted immunotherapy as a treatment for MS, particularly the adoptive transfer of *in vitro*-expanded autologous EBV-specific CD8+ T cells directed against viral latent proteins.^183^ In an open-label trial of subjects with progressive MS who received escalating doses of *in vitro*-expanded autologous EBV-specific T cells targeting EBNA1, LMP1 and LMP2A, 7 of 10 showed improvement.^185^ This approach has been explored using the adoptive transfer of partially MHC-matched allogenic EBV-specific T cells (Atara Bio, Tevogen)^186^ (clinicaltrials.gov NCT03283826). However, Atara Bio recently announced that their phase 2 EMBOLD trial of ATA188 in non-active progressive MS was negative.^187^ Whether this trial failed because of the poor survival of these allogeneic cells is unknown.^188^ Other possibilities include poor trial design, i.e. targeting people with disease that is too advanced or expecting a read-out within 12 months.^188^

Another approach is the use of an EBV vaccination to boost the immune responses to EBV. This is being explored via the use of mRNA vaccines (Moderna). An alternative means to do this could be via the use of an immune reconstitution therapy such as autologous haematopoietic stem cell transplantation (AHSCT) or alemtuzumab. The depletion-reconstitution cycle of an immune reconstitution therapy may either reduce EBV viral loads by depleting EBV-infected B cells or alternatively have a paradoxical effect via EBV reactivation, which then rejuvenates jaded, exhausted or ineffective EBV-specific T-cell responses,^189^ enabling them to control EBV.^190^ Notably, almost all MS patients undergoing AHSCT have EBV viraemia detected in the peripheral blood,^191^ which supports the latter mechanism. This high incidence of EBV viraemia further indicates that people with MS may control EBV less well than other patient groups.

## Study design

Developing and testing EBV-antiviral strategies in MS is challenging because many licensed DMTs may have anti-EBV effects, and some commentators consider it unethical to do placebo-controlled trials in people with active MS.^192,193^ Despite these considerations, it is important to have a framework for showing proof of biology, i.e. that an EBV-targeted therapy can suppress EBV latent-lytic cycling and/or reduce viral loads in that compartment. EBV biomarkers, including direct viral or indirect immunological markers, can be linked to MS disease activity in standard phase 2 and 3 MS trial designs. Proof-of-biology trials need to assess the efficacy of anti-EBV strategies against EBV and then associate this effect with a treatment effect on MS pathology, which includes both relapse biology (relapse and focal MRI activity) and smouldering associated worsening or progression independent of relapse activity (PIRA).^194^  Supplementary Table 2 summarizes some potential EBV biomarkers that could be included in clinical trials.

## Future directions

Future directions for proving EBV's causal role in MS include conducting large-scale, long-term studies that initially follow EBV-negative individuals and monitor their immunological health over time to see what happens after asymptomatic or symptomatic EBV seroconversion. This will uncover the mechanisms by which EBV might trigger MS and autoimmunity in general. Working out what happens during infectious mononucleosis and how it might affect the CNS is critical for identifying new therapeutic strategies. Developing new animal models that try to mimic the development of MS and the role of EBV or related herpes virus will help with mechanistic studies. A good start would be to extend research into Japanese macaque encephalomyelitis, a spontaneous MS-like disease in a non-human primate caused by a gamma herpes virus.^195^ This would allow researchers to study the effects of EBV infection and potential antiviral therapies in a controlled environment. Other strategies include investigating the role of EBV viral load and lytic activity in MS patients and correlating it with clinical, radiological and other biomarkers of disease activity; executing well-designed clinical trials to test the efficacy of EBV antiviral drugs and EBV-targeted immunotherapies in MS patients, as highlighted in this review; exploring a personalized medicine approach, where an individual's EBV status, viral load and other factors are studied to help identify patients who might benefit from antiviral therapies; investigating the potential of combining antiviral therapies with other MS treatments; developing novel antiviral agents that target EBV and have a favourable safety profile; and identifying biomarkers that can predict the response to antiviral therapies, which would help to guide treatment decisions and avoid unnecessary exposure to ineffective drugs.

## Conclusion

In conclusion, from an epidemiological perspective, EBV is strongly associated with MS, and its role is likely causal. Immunological, clinical and pathological studies suggest that EBV may drive MS disease activity through latent lytic infection cycling. However, robust evidence suggesting a single mechanism through which this might occur is missing. There is a strong case for testing anti-EBV strategies as a treatment for MS. This includes CNS penetrant small molecule anti-viral agents, B-cell targeting therapies and EBV-targeted immunotherapy. It is also appropriate to reassess the mode of action of currently licensed MS DMTs and therapies in late-stage development to examine how they impact EBV biology. If EBV latent-lytic cycling is driving MS disease activity, all of the licensed MS DMTs will have to show an impact on EBV biology.

## Supplementary Material

awaf170_Supplementary_Data
