# Supplementary material for: The case for targeting latent and lytic Epstein-Barr virus infection in multiple sclerosis
Source: Brain. 2025 May 6;148(9):3057–71. doi: 10.1093/brain/awaf170 (PMC12404723; doi:10.1093/brain/awaf170)
Supplement: awaf170_Supplementary_Data [file awaf170_supplementary_data.zip › awaf170_Supplementary_Data.pdf]

Supplementary Table 1 - Potential EBV-targeted therapies

Anti-EBV strategy	Class of therapy	Agents	Comment
**Latent infection**			
**B-cell targeted therapies**			
	Selective B-cell-depleting monoclonal antibodies	Anti-CD20 (rituximab, ocrelizumab, ofatumumab, ublituximab, …)<br>Anti-CD19 (inebilizumab, …)	Selective depletion of peripheral blood B-cells, variable depletion of deep tissue B-cells and unlikely to clear CNS resident pathogenic B-cells.
		Bispecific monoclonal anti-CD20/CD3 antibodies (mosunetuzumab, glofitamab, …)	Non-selective and depletes all B-cells regardless of EBV status.
		Brain shuttle CD20 inhibitor (RG6035)	Uses transferrin transporter to increase the concentration of antibody within the CNS [109–112,204–207]
	Immune reconstitution therapies	Mitoxantrone, alemtuzumab, cladribine, AHSCT, high-dose cyclophosphamide, …..	Non-selective peripheral lymphocyte depletion, including the B-cell population. Tend to be non-CNS penetrant except for cladribine.<br><br>Hypothesised that EBV-targeted cytotoxic T-lymphocyte responses may be rejuvenated post-immune reconstitution. [208–213]
	CD19 targeted CAR-T	Axicabtagene ciloleucel, tisagenlecleucel,	Selective depletion of CD19 expressing B-cells and plasmablasts. Based

	cells	lisocabtagene maraleucel, brexucabtagene autoleucel, …	on results in refractory lymphoma, this strategy has good deep tissue and CNS penetration. It is likely to be more effective than B-cell-targeted monoclonal antibodies that have poor CNS penetration. <br>Several clinical trials have started in multiple sclerosis. [214]
	Bruton Tyrosine Kinase (BTK) inhibitors	Evobrutinib, Tolebrutinib, Fenebrutinb, Remibrutinib, Orelabrutinib, GB7208, …	Ibrutinib, a first-generation BTK inhibitor, has been shown to reduce EBV viral loads in vivo and in vitro. This is likely a class effect as EBV's LMP2a signals via BTK to bypass B-cell receptor signalling, providing a pro-survival signal to EBV-infected B-cells. [157,215]
**Non-cell targeted therapies**			
	EBNA1 inhibitors	VK2019, peptide inhibitors (JLP2), …	Will target both latent and lytic-infected B-cells. A good theoretical target, but have yet to be tested in autoimmune diseases and multiple sclerosis. CNS penetration may be necessary. [216,217]
	HDAC (histone deacetylases) inhibitors	Nantinostat, chidamide, ….	HDAC inhibitors drive latent EBV to become lytic and will need to be in combination with antivirals targeting lytic infection. This class of therapies have yet to be tried in autoimmune diseases. [218,219]
	DNMT (DNA methyltransferases)	Decitabine	DNMT inhibitors induce the synthesis of LMP1, EBNA2, and EBNA3C. They could theoretically sensitise cells to lysis by EBV-specific cytotoxic

	inhibitors		T-lymphocytes (CTLs). May need to be used in combination with EBV-targeted immunotherapies. This class of therapies have yet to be tried in autoimmune diseases. [159,220]
**Antivirals targeting lytic infection**			
	DNA polymerase inhibitors	Acyclovir/valacyclovir, penciclovir/famciclovir, ganciclovir/valganciclovir, omaciclovir/valomaciclovir, cidofovir/brincidofovir, cyclopropavir, foscarnet ….	Trial results from first-generation viral DNA polymerase inhibitors were negative, with moderate activity against EBV. However, a good case exists for testing newer, more effective DNA polymerase inhibitors in MS and other autoimmune diseases. [164,221–230]
	Non-nucleoside inhibitors of viral DNA polymerase	Oxazolidinones	Biochemical and broad-spectrum cellular activity against herpes virus infections. [231]
	Antiretrovirals	Zidovudine, tenofovir disoproxil fumarate (TDF), tenofovir alafenamide (TAF), ….	Anecdotal evidence shows that this class of therapy may be effective as a disease-modifying therapy in MS. This, with in vitro data showing that these agents have anti-EBV activity, has catalysed some exploratory studies in MS. [62,68,219,232]

	Other antivirals	Artesunate, maribavir, L-dioxolane thymidine derivatives (KAY-2-41 and KAH-39-149), teriflunomide/leflunomide and other dihydroorotate dehydrogenase inhibitors, ….	There are a large number of other potential small molecule drugs that are potentially active against EBV that have the potential to be tried in multiple sclerosis. [128,131,233–235]
	Monoclonal antibodies	Anti-GP350, anti-GP350/CD89, …..	EBV antigen-specific neutralising monoclonal antibodies targeting EBV lytic infection are unlikely to prevent EBV viral reactivation but will likely prevent reinfection of naive EBV-negative B cells. Therefore, this strategy may need to be combined with other anti-EBV targeted therapies, such as an induction-maintenance strategy. [236,237]
**EBV immunotherapies targeting latent and lytic infection**			
	Autologous and allogeneic EBV-targeted T-cell therapies	Tabelecleucel, ATA188, ….	These cellular therapies are based on preliminary open-label studies of autologous EBV-specific cytotoxic T-cells. Please note a phase 2 trial of ATA188 in progressive MS was negative. [184,238]
	Therapeutic EBV vaccine	mRNA component vaccines	Based on the theory of a dysfunctional or senescent EBV-specific cytotoxic T-cell response results in poor control of EBV in patients with MS. Vaccines covering both latent and lytic EBV antigens will boost anti-EBV immunity and potentially controlling the virus.

		There is a theoretical risk that a EBV therapeutic vaccination may trigger MS disease activity via molecular mimicry. [239]
EBV-antigen-targeted CAR-T cells	GP350 and EBNA-1	Using EBV antigen-specific CAR T-cells will likely require EBV antigens to be expressed on the surface of infected cells. Because latent EBV proteins are intracellular antigens and EBV's role in driving MS is likely to be intermittent lytic re-activation, this strategy is unlikely to be effective unless autologous EBV-targeted CAR T-cells persist in vivo. The current evidence suggests that CAR T-cells don't persist long-term. [236]
Checkpoint inhibitors		The rationale is to use checkpoint inhibitors in combination with EBV immunotherapies to overcome T-cell exhaustion or senescence to boost antigen-specific T-cell responses. This is analogous to what has been tested in patients with various malignancies receiving therapeutic vaccines [240].

Supplementary Table 2 - Potential EBV-targeted therapies

Anti-EBV strategy	Class of therapy	Agents	Comment
**Latent infection**			
**B-cell targeted therapies**			
	Selective B-cell-depleting monoclonal antibodies	Anti-CD20 (rituximab, ocrelizumab, ofatumumab, ublituximab, …)<br>Anti-CD19 (inebilizumab, …)<br>Bispecific monoclonal anti-CD20/CD3 antibodies (mosunetuzumab, glofitamab, …)<br>Brain shuttle CD20 inhibitor (RG6035)	Selective depletion of peripheral blood B-cells, variable depletion of deep tissue B-cells and unlikely to clear CNS resident pathogenic B-cells.<br><br>Non-selective and depletes all B-cells regardless of EBV status.<br><br>Uses transferrin transporter to increase the concentration of antibody within the CNS [1–8]
	Immune reconstitution therapies	Mitoxantrone, alemtuzumab, cladribine, AHSCT, high-dose cyclophosphamide, …..	Non-selective peripheral lymphocyte depletion, including the B-cell population. Tend to be non-CNS penetrant except for cladribine.<br><br>Hypothesised that EBV-targeted cytotoxic T-lymphocyte responses may be rejuvenated post-immune reconstitution. [9–14]
	CD19 targeted CAR-T cells	Axicabtagene ciloleucel, tisagenlecleucel, lisocabtagene maraleucel, brexucabtagene autoleucel, …	Selective depletion of CD19 expressing B-cells and plasmablasts. Based on results in refractory lymphoma, this strategy has good deep tissue and CNS penetration. It is likely to be more effective than B-cell-targeted monoclonal antibodies that have poor CNS penetration.<br><br>Several clinical trials have started in multiple sclerosis. [15]

	Bruton Tyrosine Kinase (BTK) inhibitors	Evobrutinib, Tolebrutinib, Fenebrutinb, Remibrutinib, Orelabrutinib, GB7208, …	Ibrutinib, a first-generation BTK inhibitor, has been shown to reduce EBV viral loads in vivo and in vitro. This is likely a class effect as EBV's LMP2a signals via BTK to bypass B-cell receptor signalling, providing a pro-survival signal to EBV-infected B-cells. [16,17]
Non-cell targeted therapies			
	EBNA1 inhibitors	VK2019, peptide inhibitors (JLP2), …	Will target both latent and lytic-infected B-cells. A good theoretical target, but have yet to be tested in autoimmune diseases and multiple sclerosis. CNS penetration may be necessary. [18,19]
	HDAC (histone deacetylases) inhibitors	Nantinostat, chidamide, ….	HDAC inhibitors drive latent EBV to become lytic and will need to be in combination with antivirals targeting lytic infection. This class of therapies have yet to be tried in autoimmune diseases. [20,21]
	DNMT (DNA methyltransferases) inhibitors	Decitabine	DNMT inhibitors induce the synthesis of LMP1, EBNA2, and EBNA3C. They could theoretically sensitise cells to lysis by EBV-specific cytotoxic T-lymphocytes (CTLs). May need to be used in combination with EBV-targeted immunotherapies. This class of therapies have yet to be tried in autoimmune diseases. [22,23]
Antivirals targeting lytic infection			
	DNA polymerase inhibitors	Acyclovir/valacyclovir, penciclovir/famciclovir, ganciclovir/valganciclovir, omaciclovir/valomaciclovir, cidofovir/brincidofovir, cyclopropavir, foscarnet ….	Trial results from first-generation viral DNA polymerase inhibitors were negative, with moderate activity against EBV. However, a good case exists for testing newer, more effective DNA polymerase inhibitors in MS and other autoimmune diseases. [24–34]

	Non-nucleoside inhibitors of viral DNA polymerase	Oxazolidinones	Biochemical and broad-spectrum cellular activity against herpes virus infections. [35]
	Antiretrovirals	Zidovudine, tenofovir disoproxil fumarate (TDF), tenofovir alafenamide (TAF), ….	Anecdotal evidence shows that this class of therapy may be effective as a disease-modifying therapy in MS. This, with in vitro data showing that these agents have anti-EBV activity, has catalysed some exploratory studies in MS. [21,36–38]
	Other antivirals	Artesunate, maribavir, L-dioxolane thymidine derivatives (KAY-2-41 and KAH-39-149), teriflunomide/leflunomide and other dihydroorotate dehydrogenase inhibitors, ….	There are a large number of other potential small molecule drugs that are potentially active against EBV that have the potential to be tried in multiple sclerosis. [39–43]
	Monoclonal antibodies	Anti-GP350, anti-GP350/CD89, …..	EBV antigen-specific neutralising monoclonal antibodies targeting EBV lytic infection are unlikely to prevent EBV viral reactivation but will likely prevent reinfection of naive EBV-negative B cells. Therefore, this strategy may need to be combined with other anti-EBV targeted therapies, such as an induction-maintenance strategy. [44,45]
EBV immunotherapies targeting latent and lytic infection			
	Autologous and allogeneic EBV-targeted T-cell therapies	Tabelecleucel, ATA188, ….	These cellular therapies are based on preliminary open-label studies of autologous EBV-specific cytotoxic T-cells. Please note a phase 2 trial of ATA188 in progressive MS was negative. [46,47]
	Therapeutic EBV vaccine	mRNA component vaccines	Based on the theory of a dysfunctional or senescent EBV-specific cytotoxic T-cell response results in poor control of EBV in patients with MS. Vaccines covering both latent and lytic EBV antigens will boost anti-EBV immunity and potentially controlling the virus.<br><br>There is a theoretical risk that a EBV therapeutic vaccination may trigger MS disease

			activity via molecular mimicry. [48]
	EBV-antigen-targeted CAR-T cells	GP350 and EBNA-1	Using EBV antigen-specific CAR T-cells will likely require EBV antigens to be expressed on the surface of infected cells. Because latent EBV proteins are intracellular antigens and EBV's role in driving MS is likely to be intermittent lytic re-activation, this strategy is unlikely to be effective unless autologous EBV-targeted CAR T-cells persist in vivo. The current evidence suggests that CAR T-cells don't persist long-term. [44]
	Checkpoint inhibitors		The rationale is to use checkpoint inhibitors in combination with EBV immunotherapies to overcome T-cell exhaustion or senescence to boost antigen-specific T-cell responses. This is analogous to what has been tested in patients with various malignancies receiving therapeutic vaccines [49].

Supplementary Table 3: EBV-associated biomarkers that could potentially be used in proof-of-biology trials targeting EBV

**Class of biomarker**	**Biomarker**	**Comments and references**
**EBV infection (latent and lytic)**		
EBV viral loads	Peripheral blood quantitative real-time PCR (qPCR) - whole blood, plasma or cells (peripheral blood mononuclear cells (PBMCs), B-cells-CD19+, memory B-cells-CD19+/CD27+)	Standard assay for detecting EBV viral loads. It does not differentiate between latent or lytic infection. However, high plasma (cell-free) viral loads imply lytic infection [50].
	qPCR - cerebrospinal	In general, standard quantitative real-time PCR (qPCR) is used to detect EBV DNA in CSF [51,52]
	Digital droplet PCR (ddPCR)	ddPCE is a technique that combines PCR and droplet microfluidics to perform amplification reactions in droplets and is more sensitive than standard qPCR[53]
**Lytic infection**		
	Peripheral blood qRT-PCR - whole blood, plasma or PBMCs	Standard qPCR is used to detect EBV DNA in whole blood, plasma or PBMCs[54]
	qRT-PCR EBV DNA in saliva	Standard qPCR is used to detect EBV DNA in saliva[55]
	BZLF1 (EBV ZEBRA protein) mRNA detection saliva	BZLF1 is a basic leucine zipper transcriptional activator required for EBV latent to lytic reactivation [56]
**Latent infection**		
	EBER+ cells using FACs	EBV encodes small nonpolyadenylated, non-coding (nc) RNAs called EBV-encoded RNA (EBER) are the most abundant viral transcripts in latently EBV-infected cells[57]

	PBMCs EBER levels	Quantitative assay to detect EBER transcripts in PBMCs[58,59]
	Exosomal EBV-microRNAs	Exosomal EBV-microRNAs are microRNAs (miRNAs) are transported from infected cells to other cells via exosomes[58,59]
	Exosomal EBNA1, LMP1 and LMP2A proteins	Exosomal EBNA1, LMP1 and LMP2A are latent EBV proteins that are found on exosomes[60]
**Potential immunological biomarkers**		
	Antibody titres to latent and lytic EBV proteins	Quantitative assays to detect specific antibody titres or levels to latent and lytic EBV proteins[61]
	EBV-specific T-cell repertoire (TCR)analysis	Semi-quantitative assays to detect specific TCRs that bind peptides in derived from latent and lytic EBV proteins. The TCR-usage is HLA-dependent [62,63]

Supplementary References

1. RG-6035 by Genentech USA for Primary Progressive Multiple Sclerosis (PPMS): Likelihood of Approval. Pharmaceutical Technology. January 5, 2024. Accessed December 6, 2024. http://www.pharmaceutical-technology.com/data-insights/rg-6035-genentech-usa-primary-progressive-multiple-sclerosis-ppms-likelihood-of-approval/

2. Hauser SL, Waubant E, Arnold DL, et al. B-cell depletion with rituximab in relapsing-remitting multiple sclerosis. *N Engl J Med*. 2008;358(7):676-688.

3. Hauser SL, Bar-Or A, Comi G, et al. Ocrelizumab versus Interferon Beta-1a in Relapsing Multiple Sclerosis. *N Engl J Med*. 2017;376(3):221-234.

4. Montalban X, Hauser SL, Kappos L, et al. Ocrelizumab versus Placebo in Primary Progressive Multiple Sclerosis. *N Engl J Med*. 2017;376(3):209-220.

5. Hauser SL, Bar-Or A, Cohen JA, et al. Ofatumumab versus Teriflunomide in Multiple Sclerosis. *N Engl J Med*. 2020;383(6):546-557.

6. Steinman L, Fox E, Hartung HP, et al. Ublituximab versus Teriflunomide in Relapsing Multiple Sclerosis. *N Engl J Med*. 2022;387(8):704-714.

7. Yan L, Kimko H, Wang B, Cimbora D, Katz E, Rees WA. Population Pharmacokinetic Modeling of Inebilizumab in Subjects with Neuromyelitis Optica Spectrum Disorders, Systemic Sclerosis, or Relapsing Multiple Sclerosis. *Clin Pharmacokinet*. 2022;61(3):387-400.

8. Shah K, Leandro M, Cragg M, et al. Disrupting B and T-cell collaboration in autoimmune disease: T-cell engagers versus CAR T-cell therapy? *Clin Exp Immunol*. 2024;217(1):15-30.

9. Hartung HP, Gonsette R, König N, et al. Mitoxantrone in progressive multiple sclerosis: a placebo-controlled, double-blind, randomised, multicentre trial. *Lancet*. 2002;360(9350):2018-2025.

10. Coles AJ, Twyman CL, Arnold DL, et al. Alemtuzumab for patients with relapsing multiple sclerosis after disease-modifying therapy: a randomised controlled phase 3 trial. *Lancet*. 2012;380(9856):1829-1839.

11. Cohen JA, Coles AJ, Arnold DL, et al. Alemtuzumab versus interferon beta 1a as first-line treatment for patients with relapsing-remitting multiple sclerosis: a randomised controlled phase 3 trial. *Lancet*. 2012;380(9856):1819-1828.

12. Giovannoni G, Comi G, Cook S, et al. A placebo-controlled trial of oral cladribine for relapsing multiple sclerosis. *N Engl J Med*. 2010;362(5):416-426.

13. Muraro PA, Martin R, Mancardi GL, Nicholas R, Sormani MP, Saccardi R. Autologous haematopoietic stem cell transplantation for treatment of multiple sclerosis. *Nat Rev Neurol*. 2017;13(7):391-405.

14. Gladstone DE, Zamkoff KW, Krupp L, et al. High-dose cyclophosphamide for moderate to severe refractory multiple sclerosis. *Arch Neurol*. 2006;63(10):1388-1393.

15. Schett G, Mackensen A, Mougiakakos D. CAR T-cell therapy in autoimmune diseases. *Lancet*. 2023;402(10416):2034-2044.

16. Krämer J, Bar-Or A, Turner TJ, Wiendl H. Bruton tyrosine kinase inhibitors for multiple sclerosis. *Nat Rev Neurol*. 2023;19(5):289-304.

17. Singh DR, Nelson SE, Pawelski AS, et al. Epstein-Barr virus LMP1 protein promotes proliferation and inhibits differentiation of epithelial cells via activation of YAP and TAZ. *Proc Natl Acad Sci U S A*. 2023;120(20):e2219755120.

18. Davis MT, Anders NM, Colevas AD, Messick TE, Rudek MA. Validation of a robust and rapid liquid chromatography tandem mass spectrometric method for the quantitative analysis of VK-2019, a selective EBNA1 inhibitor. *Biomed Chromatogr*. 2024;38(2):e5775.

19. Jiang L, Lui YL, Li H, et al. EBNA1-specific luminescent small molecules for the imaging and inhibition of latent EBV-infected tumor cells. *Chem Commun (Camb)*. 2014;50(49):6517-6519.

20. Haverkos B, Alpdogan O, Baiocchi R, et al. Targeted therapy with nanatinostat and valganciclovir in recurrent EBV-positive lymphoid malignancies: a phase 1b/2 study. *Blood Adv*. 2023;7(20):6339-6350.

21. Xu L, Zhang M, Tu D, et al. Chidamide Induces Epstein-Barr Virus (EBV) Lytic Infection and Acts Synergistically with Tenofovir to Eliminate EBV-Positive Burkitt Lymphoma. *J Pharmacol Exp Ther*. 2023;387(3):288-298.

22. Preston-Alp S, Caruso LB, Su C, et al. Decitabine disrupts EBV genomic epiallele DNA methylation patterns around CTCF binding sites to increase chromatin accessibility and lytic transcription in gastric cancer. *mBio*. 2023;14(5):e0039623.

23. Dalton T, Doubrovina E, Pankov D, et al. Epigenetic reprogramming sensitizes immunologically silent EBV+ lymphomas to virus-directed immunotherapy. *Blood*. 2020;135(21):1870-1881.

24. Pagano JS, Sixbey JW, Lin JC. Acyclovir and Epstein-Barr virus infection. *J Antimicrob Chemother*. 1983;12 Suppl B:113-121.

25. Bacon TH, Boyd MR. Activity of penciclovir against Epstein-Barr virus. *Antimicrob Agents Chemother*. 1995;39(7):1599-1602.

26. Goldani LZ. Treatment of severe infectious mononucleosis with famciclovir. *J Infect*. 2002;44(2):92-93.

27. Meng Q, Hagemeier SR, Fingeroth JD, Gershburg E, Pagano JS, Kenney SC. The Epstein-Barr virus (EBV)-encoded protein kinase, EBV-PK, but not the thymidine kinase (EBV-TK), is required for ganciclovir and acyclovir inhibition of lytic viral production. *J Virol*. 2010;84(9):4534-4542.

28. Albatati S, Sharma A, Haubrich K, Wright A, Gantt S, Blydt-Hansen TD. Valganciclovir prophylaxis delays onset of EBV viremia in high-risk pediatric solid organ transplant recipients. *Pediatr Res*. 2020;87(5):892-896.

29. Hoshino Y, Katano H, Zou P, et al. Long-term administration of valacyclovir reduces the number of Epstein-Barr virus (EBV)-infected B cells but not the number of EBV DNA copies per B cell in healthy

volunteers. *J Virol.* 2009;83(22):11857-11861.

30. De Paor M, O'Brien K, Fahey T, Smith SM. Antiviral agents for infectious mononucleosis (glandular fever). *Cochrane Database Syst Rev.* 2016;12(12):CD011487.
31. Abdulkarim B, Sabri S, Zelenika D, et al. Antiviral agent cidofovir decreases Epstein-Barr virus (EBV) oncoproteins and enhances the radiosensitivity in EBV-related malignancies. *Oncogene.* 2003;22(15):2260-2271.
32. Camargo JF, Morris MI, Abbo LM, et al. The use of brincidofovir for the treatment of mixed dsDNA viral infection. *J Clin Virol.* 2016;83:1-4.
33. Prichard MN, Williams JD, Komazin-Meredith G, et al. Synthesis and antiviral activities of methylenecyclopropane analogs with 6-alkoxy and 6-alkylthio substitutions that exhibit broad-spectrum antiviral activity against human herpesviruses. *Antimicrob Agents Chemother.* 2013;57(8):3518-3527.
34. Afshar K, Rao AP, Patel V, Forrester K, Ganesh S. Use of Foscarnet Therapy for EBV Infection following Control of PTLD with Enhancement of Cellular Immunity in a Lung-Transplant Recipient. *J Transplant.* 2011;2011:919651.
35. Plotkin MA, Labroli M, Schubert J, et al. Discovery of Broad-Spectrum Herpes Antiviral Oxazolidinone Amide Derivatives and Their Structure-Activity Relationships. *ACS Med Chem Lett.* 2024;15(8):1232-1241.
36. Bayraktar UD, Diaz LA, Ashlock B, et al. Zidovudine-based lytic-inducing chemotherapy for Epstein-Barr virus-related lymphomas. *Leuk Lymphoma.* 2014;55(4):786-794.
37. Drosu NC, Edelman ER, Housman DE. Tenofovir prodrugs potently inhibit Epstein-Barr virus lytic DNA replication by targeting the viral DNA polymerase. *Proc Natl Acad Sci U S A.* 2020;117(22):12368-12374.
38. Torkildsen Ø, Myhr KM, Skogen V, Steffensen LH, Bjørnevik K. Tenofovir as a treatment option for multiple sclerosis. *Mult Scler Relat Disord.* 2020;46:102569.
39. Auerochs S, Korn K, Marschall M. A reporter system for Epstein-Barr virus (EBV) lytic replication: anti-EBV activity of the broad anti-herpesviral drug artesunate. *J Virol Methods.* 2011;173(2):334-339.
40. Whitehurst CB, Sanders MK, Law M, et al. Maribavir inhibits Epstein-Barr virus transcription through the EBV protein kinase. *J Virol.* 2013;87(9):5311-5315.
41. Coen N, Duraffour S, Haraguchi K, et al. Antiherpesvirus activities of two novel 4'-thiothymidine derivatives, KAY-2-41 and KAH-39-149, are dependent on viral and cellular thymidine kinases. *Antimicrob Agents Chemother.* 2014;58(8):4328-4340.
42. Bilger A, Plowshay J, Ma S, et al. Leflunomide/teriflunomide inhibit Epstein-Barr virus (EBV)- induced lymphoproliferative disease and lytic viral replication. *Oncotarget.* 2017;8(27):44266-44280.
43. Gold J, Holden D, Parratt J, et al. Effect of teriflunomide on Epstein-Barr virus shedding in relapsing-remitting multiple sclerosis patients: Outcomes from a real-world pilot cohort study. *Mult Scler Relat Disord.* 2022;68:104377.
44. Zhang X, Wang T, Zhu X, et al. GMP development and preclinical validation of CAR-T cells targeting a lytic EBV antigen for therapy of EBV-associated malignancies. *Front Immunol.* 2023;14:1103695.
45. Sokal EM, Hoppenbrouwers K, Vandermeulen C, et al. Recombinant gp350 vaccine for infectious mononucleosis: a phase 2, randomized, double-blind, placebo-controlled trial to evaluate the safety, immunogenicity, and efficacy of an Epstein-Barr virus vaccine in healthy young adults. *J Infect Dis.* 2007;196(12):1749-1753.
46. Keam SJ. Tabelecleucel: First Approval. *Mol Diagn Ther.* 2023;27(3):425-431.
47. Giovannoni G, Hawkes CH, Lechner-Scott J, Levy M, Yeh EA. Emboldened or not: The potential fall-out of a failed anti-EBV trial in multiple sclerosis. *Mult Scler Relat Disord.* 2023;81:105364.
48. Cui X, Snapper CM. Epstein Barr Virus: Development of Vaccines and Immune Cell Therapy for EBV-Associated Diseases. *Front Immunol.* 2021;12:734471.

49. Carvalho T. Personalized anti-cancer vaccine combining mRNA and immunotherapy tested in melanoma trial. *Nat Med*. 2023;29(10):2379-2380.

50. Gulley ML, Tang W. Using Epstein-Barr viral load assays to diagnose, monitor, and prevent posttransplant lymphoproliferative disorder. *Clin Microbiol Rev*. 2010;23(2):350-366.

51. Weinberg A, Li S, Palmer M, Tyler KL. Quantitative CSF PCR in Epstein-Barr virus infections of the central nervous system. *Ann Neurol*. 2002;52(5):543-548.

52. Wang Y, Yang J, Wen Y. Lessons from Epstein-Barr virus DNA detection in cerebrospinal fluid as a diagnostic tool for EBV-induced central nervous system dysfunction among HIV-positive patients. *Biomed Pharmacother*. 2022;145:112392.

53. Soldan SS, Su C, Monaco MC, et al. Multiple sclerosis patient-derived spontaneous B cells have distinct EBV and host gene expression profiles in active disease. *Nat Microbiol*. 2024;9(6):1540-1554.

54. Vinuesa V, Solano C, Giménez E, Navarro D. Comparison of the artus Epstein-Barr virus (EBV) PCR kit and the Abbott RealTime EBV assay for measuring plasma EBV DNA loads in allogeneic stem cell transplant recipients. *Diagn Microbiol Infect Dis*. 2017;88(1):36-38.

55. Holden DW, Gold J, Hawkes CH, et al. Epstein Barr virus shedding in multiple sclerosis: Similar frequencies of EBV in saliva across separate patient cohorts. *Mult Scler Relat Disord*. 2018;25:197-199.

56. Fagin U, Nerbas L, Vogl B, Jabs WJ. Analysis of BZLF1 mRNA detection in saliva as a marker for active replication of Epstein-Barr virus. *J Virol Methods*. 2017;244:11-16.

57. Shannon-Lowe C, Adland E, Bell AI, Delecluse HJ, Rickinson AB, Rowe M. Features distinguishing Epstein-Barr virus infections of epithelial cells and B cells: viral genome expression, genome maintenance, and genome amplification. *J Virol*. 2009;83(15):7749-7760.

58. Baglio SR, van Eijndhoven MAJ, Koppers-Lalic D, et al. Sensing of latent EBV infection through exosomal transfer of 5'pppRNA. *Proc Natl Acad Sci U S A*. 2016;113(5):E587-E596.

59. Mohammadinasr M, Montazersaheb S, Hosseini V, et al. Epstein-Barr virus-encoded BART9 and BART15 miRNAs are elevated in exosomes of cerebrospinal fluid from relapsing-remitting multiple sclerosis patients. *Cytokine*. 2024;179:156624.

60. Mrad MF, Saba ES, Nakib L, Khoury SJ. Exosomes From Subjects With Multiple Sclerosis Express EBV-Derived Proteins and Activate Monocyte-Derived Macrophages. *Neurol Neuroimmunol Neuroinflamm*. 2021;8(4). doi:10.1212/NXI.0000000000001004

61. Persson Berg L, Eriksson M, Longhi S, et al. Serum IgG levels to Epstein-Barr and measles viruses in patients with multiple sclerosis during natalizumab and interferon beta treatment. *BMJ Neurol Open*. 2022;4(2):e000271.

62. Schneider-Hohendorf T, Gerdes LA, Pignolet B, et al. Broader Epstein-Barr virus-specific T cell receptor repertoire in patients with multiple sclerosis. *J Exp Med*. 2022;219(11). doi:10.1084/jem.20220650

63. Schneider-Hohendorf T, Wünsch C, Falk S, et al. Broader anti-EBV TCR repertoire in multiple sclerosis: disease specificity and treatment modulation. *Brain*. Published online July 18, 2024. doi:10.1093/brain/awae244
