# Supplementary material for: The case for targeting latent and lytic Epstein-Barr virus infection in multiple sclerosis
Source: Brain. 2025 May 6;148(9):3057–71. doi: 10.1093/brain/awaf170 (PMC12404723; doi:10.1093/brain/awaf170)
Supplement: awaf170_Supplementary_Data [file awaf170_supplementary_data.zip › brain-2024-03099-File006.pdf]

Supplementary Table 1 - Potential EBV-targeted therapies

Anti-EBV strategy	Class of therapy	Agents	Comment
**Latent infection**			
**B-cell targeted therapies**			
	Selective B-cell-depleting monoclonal antibodies	Anti-CD20 (rituximab, ocrelizumab, ofatumumab, ublituximab, …)<br>Anti-CD19 (inebilizumab, …)	Selective depletion of peripheral blood B-cells, variable depletion of deep tissue B-cells and unlikely to clear CNS resident pathogenic B-cells.
		Bispecific monoclonal anti-CD20/CD3 antibodies (mosunetuzumab, glofitamab, …)	Non-selective and depletes all B-cells regardless of EBV status.
		Brain shuttle CD20 inhibitor (RG6035)	Uses transferrin transporter to increase the concentration of antibody within the CNS [109–112,204–207]
	Immune reconstitution therapies	Mitoxantrone, alemtuzumab, cladribine, AHSCT, high-dose cyclophosphamide, …..	Non-selective peripheral lymphocyte depletion, including the B-cell population. Tend to be non-CNS penetrant except for cladribine.<br><br>Hypothesised that EBV-targeted cytotoxic T-lymphocyte responses may be rejuvenated post-immune reconstitution. [208–213]
	CD19 targeted CAR-T	Axicabtagene ciloleucel, tisagenlecleucel,	Selective depletion of CD19 expressing B-cells and plasmablasts. Based

	cells	lisocabtagene maraleucel, brexucabtagene autoleucel, …	on results in refractory lymphoma, this strategy has good deep tissue and CNS penetration. It is likely to be more effective than B-cell-targeted monoclonal antibodies that have poor CNS penetration.<br><br>Several clinical trials have started in multiple sclerosis. [214]
	Bruton Tyrosine Kinase (BTK) inhibitors	Evobrutinib, Tolebrutinib, Fenebrutinb, Remibrutinib, Orelabrutinib, GB7208, …	Ibrutinib, a first-generation BTK inhibitor, has been shown to reduce EBV viral loads in vivo and in vitro. This is likely a class effect as EBV's LMP2a signals via BTK to bypass B-cell receptor signalling, providing a pro-survival signal to EBV-infected B-cells. [157,215]
**Non-cell targeted therapies**			
	EBNA1 inhibitors	VK2019, peptide inhibitors (JLP2), …	Will target both latent and lytic-infected B-cells. A good theoretical target, but have yet to be tested in autoimmune diseases and multiple sclerosis. CNS penetration may be necessary. [216,217]
	HDAC (histone deacetylases) inhibitors	Nantinostat, chidamide, ….	HDAC inhibitors drive latent EBV to become lytic and will need to be in combination with antivirals targeting lytic infection. This class of therapies have yet to be tried in autoimmune diseases. [218,219]
	DNMT (DNA methyltransferases)	Decitabine	DNMT inhibitors induce the synthesis of LMP1, EBNA2, and EBNA3C. They could theoretically sensitise cells to lysis by EBV-specific cytotoxic

	inhibitors		T-lymphocytes (CTLs). May need to be used in combination with EBV-targeted immunotherapies. This class of therapies have yet to be tried in autoimmune diseases. [159,220]
**Antivirals targeting lytic infection**			
	DNA polymerase inhibitors	Acyclovir/valacyclovir, penciclovir/famciclovir, ganciclovir/valganciclovir, omaciclovir/valomaciclovir, cidofovir/brincidofovir, cyclopropavir, foscarnet ….	Trial results from first-generation viral DNA polymerase inhibitors were negative, with moderate activity against EBV. However, a good case exists for testing newer, more effective DNA polymerase inhibitors in MS and other autoimmune diseases. [164,221–230]
	Non-nucleoside inhibitors of viral DNA polymerase	Oxazolidinones	Biochemical and broad-spectrum cellular activity against herpes virus infections. [231]
	Antiretrovirals	Zidovudine, tenofovir disoproxil fumarate (TDF), tenofovir alafenamide (TAF), ….	Anecdotal evidence shows that this class of therapy may be effective as a disease-modifying therapy in MS. This, with in vitro data showing that these agents have anti-EBV activity, has catalysed some exploratory studies in MS. [62,68,219,232]

Other antivirals	Artesunate, maribavir, L-dioxolane thymidine derivatives (KAY-2-41 and KAH-39-149), teriflunomide/leflunomide and other dihydroorotate dehydrogenase inhibitors, ….	There are a large number of other potential small molecule drugs that are potentially active against EBV that have the potential to be tried in multiple sclerosis. [128,131,233–235]
Monoclonal antibodies	Anti-GP350, anti-GP350/CD89, …..	EBV antigen-specific neutralising monoclonal antibodies targeting EBV lytic infection are unlikely to prevent EBV viral reactivation but will likely prevent reinfection of naive EBV-negative B cells. Therefore, this strategy may need to be combined with other anti-EBV targeted therapies, such as an induction-maintenance strategy. [236,237]
**EBV immunotherapies targeting latent and lytic infection**		
Autologous and allogeneic EBV-targeted T-cell therapies	Tabelecleucel, ATA188, ….	These cellular therapies are based on preliminary open-label studies of autologous EBV-specific cytotoxic T-cells. Please note a phase 2 trial of ATA188 in progressive MS was negative. [184,238]
Therapeutic EBV vaccine	mRNA component vaccines	Based on the theory of a dysfunctional or senescent EBV-specific cytotoxic T-cell response results in poor control of EBV in patients with MS. Vaccines covering both latent and lytic EBV antigens will boost anti-EBV immunity and potentially controlling the virus.

		There is a theoretical risk that a EBV therapeutic vaccination may trigger MS disease activity via molecular mimicry. [239]
EBV-antigen-targeted CAR-T cells	GP350 and EBNA-1	Using EBV antigen-specific CAR T-cells will likely require EBV antigens to be expressed on the surface of infected cells. Because latent EBV proteins are intracellular antigens and EBV's role in driving MS is likely to be intermittent lytic re-activation, this strategy is unlikely to be effective unless autologous EBV-targeted CAR T-cells persist in vivo. The current evidence suggests that CAR T-cells don't persist long-term. [236]
Checkpoint inhibitors		The rationale is to use checkpoint inhibitors in combination with EBV immunotherapies to overcome T-cell exhaustion or senescence to boost antigen-specific T-cell responses. This is analogous to what has been tested in patients with various malignancies receiving therapeutic vaccines [240].

Supplementary Table 2 Supplementary: EBV-associated biomarkers that could potentially be used in proof-of-biology trials targeting EBV

Class of biomarker	Biomarker	Comments and references
**EBV infection (latent and lytic)**		
EBV viral loads	Peripheral blood quantitative real-time PCR (qPCR) - whole blood, plasma or cells (peripheral blood mononuclear cells (PBMCs), B-cells-CD19+, memory B-cells-CD19+/CD27+)	Standard assay for detecting EBV viral loads. It does not differentiate between latent or lytic infection. However, high plasma (cell-free) viral loads imply lytic infection [191].
	qPCR - cerebrospinal	In general, standard quantitative real-time PCR (qPCR) is used to detect EBV DNA in CSF [192,193]
	Digital droplet PCR (ddPCR)	ddPCE is a technique that combines PCR and droplet microfluidics to perform amplification reactions in droplets and is more sensitive than standard qPCR[194]
**Lytic infection**		
	Peripheral blood qRT-PCR - whole blood, plasma or PBMCs	Standard qPCR is used to detect EBV DNA in whole blood, plasma or PBMCs[195]
	qRT-PCR EBV DNA in saliva	Standard qPCR is used to detect EBV DNA in saliva[196]
	BZLF1 (EBV ZEBRA protein) mRNA detection saliva	BZLF1 is a basic leucine zipper transcriptional activator required for EBV latent to lytic reactivation [197]
**Latent infection**		
	EBER+ cells using FACs	EBV encodes small nonpolyadenylated, non-coding (nc) RNAs called EBV-encoded RNA (EBER) are the most abundant viral transcripts in latently EBV-infected cells[198]
	PBMCs EBER levels	Quantitative assay to detect EBER transcripts in PBMCs[199,200]

	Exosomal EBV-microRNAs	Exosomal EBV-microRNAs are microRNAs (miRNAs) are transported from infected cells to other cells via exosomes[199,200]
	Exosomal EBNA1, LMP1 and LMP2A proteins	Exosomal EBNA1, LMP1 and LMP2A are latent EBV proteins that are found on exosomes[201]
**Potential immunological biomarkers**		
	Antibody titres to latent and lytic EBV proteins	Quantitative assays to detect specific antibody titres or levels to latent and lytic EBV proteins[103]
	EBV-specific T-cell repertoire (TCR)analysis	Semi-quantitative assays to detect specific TCRs that bind peptides in derived from latent and lytic EBV proteins. The TCR-usage is HLA-dependent [106,202]
